# The role of Frenkel defect diffusion in dynamic annealing in ion-irradiated Si

**DOI:** 10.1038/srep39754

**Published:** 2017-01-06

**Authors:** J. B. Wallace, L. B. Bayu Aji, A. A. Martin, S. J. Shin, L. Shao, S. O. Kucheyev

**Affiliations:** 1 Lawrence Livermore National Laboratory, Livermore, California 94550, USA; 2 Department of Nuclear Engineering, Texas A&M University, College Station, Texas 77843, USA.

## Abstract

The formation of stable radiation damage in crystalline solids often proceeds via complex dynamic annealing processes, involving migration and interaction of ballistically-generated point defects. The dominant dynamic annealing processes, however, remain unknown even for crystalline Si. Here, we use a pulsed ion beam method to study defect dynamics in Si bombarded in the temperature range from −20 to 140 °C with 500 keV Ar ions. Results reveal a defect relaxation time constant of ~10–0.2 ms, which decreases monotonically with increasing temperature. The dynamic annealing rate shows an Arrhenius dependence with two well-defined activation energies of 73 ± 5 meV and 420 ± 10 meV, below and above 60 °C, respectively. Rate theory modeling, bench-marked against this data, suggests a crucial role of both vacancy and interstitial diffusion, with the dynamic annealing rate limited by the migration and interaction of vacancies.

The formation of stable radiation damage in crystalline materials often proceeds via so-called dynamic annealing (DA) processes, involving migration, recombination, and clustering of mobile point defects *during* irradiation. These DA processes are complex and remain poorly understood even for crystalline Si, which is the most extensively studied and arguably best understood material^1^. Since DA is believed to be thermally activated, determining activation energies (*E*_*a*_s) of the dominant DA processes is one of the first logical steps toward understanding radiation damage dynamics.

Several attempts to understand DA in Si by measuring associated *E*_*a*_s have been reported, albeit with limited success as they have revealed a very wide range of *E*_*a*_s of ~0.2–1.7 eV^2^,^3^,^4^,^5^. For example, Linnros and Holmen^2^ have extracted an *E*_*a*_ of 1.2 eV from the temperature (*T*) dependence of the ion dose rate at which the crystalline/amorphous interface is stationary for bombardment with 1.5 MeV Ne, Ar, or Xe ions in the *T* range of ~100–300 °C. In contrast, Schultz *et al*.^3^ have found an *E*_*a*_ of 0.9 eV by plotting the *T*-dependence of the dose rate required to reach amorphization in the crystal bulk at a fixed dose for 1 MeV Si ion irradiation in a narrow *T* range of ~40–80 °C. Goldberg *et al*.^4^ have expanded the approach of Schultz *et al*.^3^ and found *E*_*a*_s in a wide range of ~0.7–1.7 eV for 80 keV ions with masses ranging from ^12^C to ^132^Xe and *T* in the range of ~10–300 °C. They^4^ have found that the *E*_*a*_ value increases close-to-linearly with either ion mass or *T* (since, with increasing ion mass, the onset of bulk amorphization occurs at higher *T*s for any given dose and dose rate). Finally, Kinomura *et al*.^5^, following the work of Linnros *et al*.^6^, have systematically studied *T* dependencies of the rate of ion-beam-induced epitaxial crystallization and found *E*_*a*_ values of ~0.3–0.4 eV in the *T* range of ~250–400 °C for irradiation with 3 MeV C, Si, Ge, or Au ions and an *E*_*a*_ of ~0.2 eV for 3 MeV C ion bombardment in the *T* range of ~150–280 °C. Such large inconsistency in the *E*_*a*_s reported (~0.2–1.7 eV) highlights the complexity and currently limited understanding of the fundamental physics governing DA even for Si.

Here, we use a novel pulsed ion beam technique^7^,^8^,^9^,^10^ to measure the *T* dependence of the DA time constant (*τ*) in Si bombarded with 500 keV Ar ions in a regime of relatively high ion doses when damage accumulation is dominated by inter-cascade DA processes (i.e., by the interaction of mobile defects generated in different collision cascades). Our results reveal two well-defined regions in the Arrhenius plot of the DA rate, with very different *E*_*a*_s of 73 and 420 meV, below and above ~60 °C, respectively. A comparison of these *E*_*a*_s with the literature values^11^,^12^,^13^,^14^,^15^ and our rate theory modeling results suggest that inter-cascade communication in Ar-ion-bombarded Si is carried out primarily by migrating interstitials and vacancies, with vacancy migration and interaction being the rate limiting processes.

## Experimental

**Figure 1:**
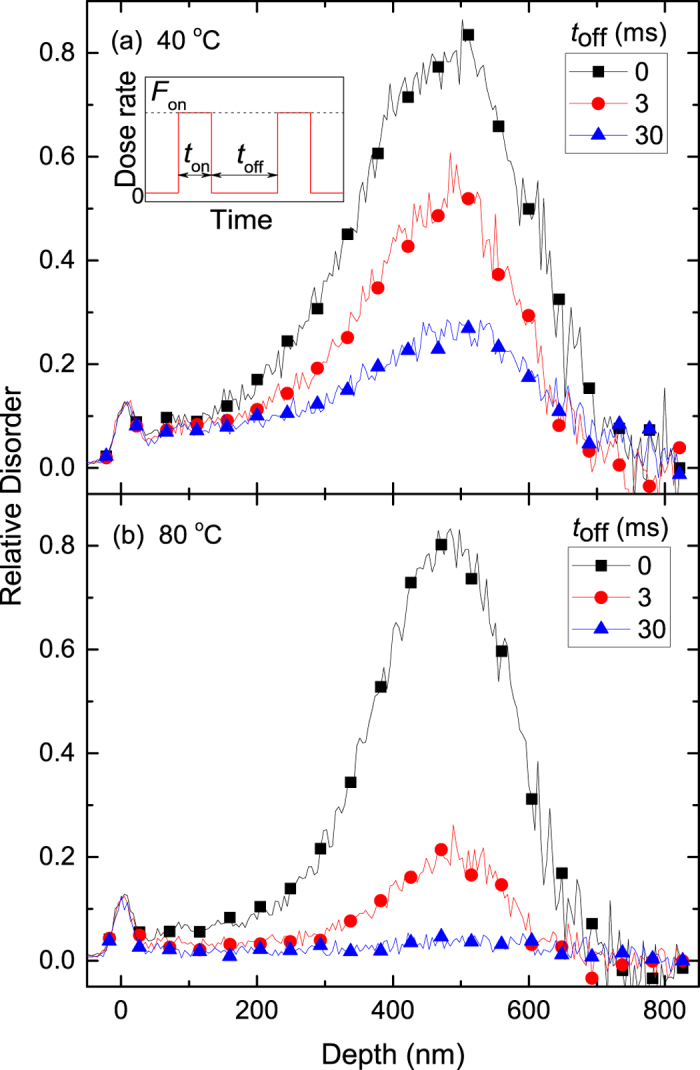
Selected depth profiles of relative disorder in Si bombarded with a pulsed beam of 500 keV Ar ions with *F*_*on*_ = 1.9 × 10^13^ cm^−2^ s^−1^, *t*_*on*_ = 1 ms, and different *t*_*off*_ values given in legends at temperatures and doses of (**a**) 40 °C and 2.7 × 10^14^ cm^−2^ and (**b**) 80 °C and 5.0 × 10^14^ cm^−2^. For clarity, only every 10th experimental point is depicted. The inset in (**a**) is a schematic of the time dependence of the instantaneous dose rate for pulsed beam irradiation, defining *t*_*on*_, *t*_*off*_, and *F*_*on*_.

The 4 MV ion accelerator (National Electrostatics Corporation, model 4UH) at Lawrence Livermore National Laboratory was used for both ion irradiation and ion beam analysis. Float-zone grown (100) Si single crystals (with a resistivity of ~5 Ω cm) were bombarded with 500 keV ^40^Ar^+^ ions at 7° off the [100] direction in the *T* range from −20 to 140 °C. To improve thermal contact, the samples were attached to the Cu sample holder with conductive Ag paste. All irradiations were performed in a broad beam mode^7^. In each irradiation run, the total dose was split into a train of equal square pulses each with an instantaneous dose rate *F*_*on*_ ≈ 1.9 × 10^13^ cm^−2^ s^−1^ and duration *t*_*on*_ = 1 ms, corresponding to ~4.6 × 10^−5^ displacements per atom per pulse. The depth profile of ballistically-generated vacancies was calculated with the TRIM code (version SRIM-2013.00)^16^ with an atomic concentration of Si of 4.98 × 10^22^ atoms cm^−3^ and a threshold energy for atomic displacements of 13 eV. The pulsing parameters *F*_*on*_ and *t*_*on*_ were chosen based on previous pulsed beam measurements of Si at room *T*^7^,^8^ in order to maximize the DA efficiency and to limit the inter-cascade defect interaction within each pulse. The adjacent pulses were separated by time *t*_*off*_, which was varied between 0.2 and 50 ms. The inset in Fig. 1(a) shows a schematic of the time dependence of the instantaneous dose rate and defines pulsing parameters *t*_*on*_, *t*_*off*_, and *F*_*on*_. A more detailed description of the experimental arrangement can be found elsewhere^7^,^8^.

The dependence of lattice damage on *t*_*off*_ was studied *ex*-*situ* at room *T* by ion channeling. Depth profiles of lattice disorder were measured with 2 MeV ^4^He^+^ ions incident along the [100] direction and backscattered into a detector at 164° relative to the incident beam direction. Raw channeling spectra were analyzed with one of the conventional algorithms^17^ for extracting depth profiles of relative disorder. Values of average relative bulk disorder (*n*) were obtained by averaging depth profiles of relative disorder over 20 channels (~38 nm) centered on the bulk damage peak maximum. Error bars of *n* are standard deviations. Total ion doses at different *T*s were different and chosen such that, for continuous beam irradiation (i.e., *t*_*off*_ = 0), *n* was in the range of 0.6–0.9 (with *n* = 1 corresponding to full amorphization).

## Results and Discussion

**Figure 2:**
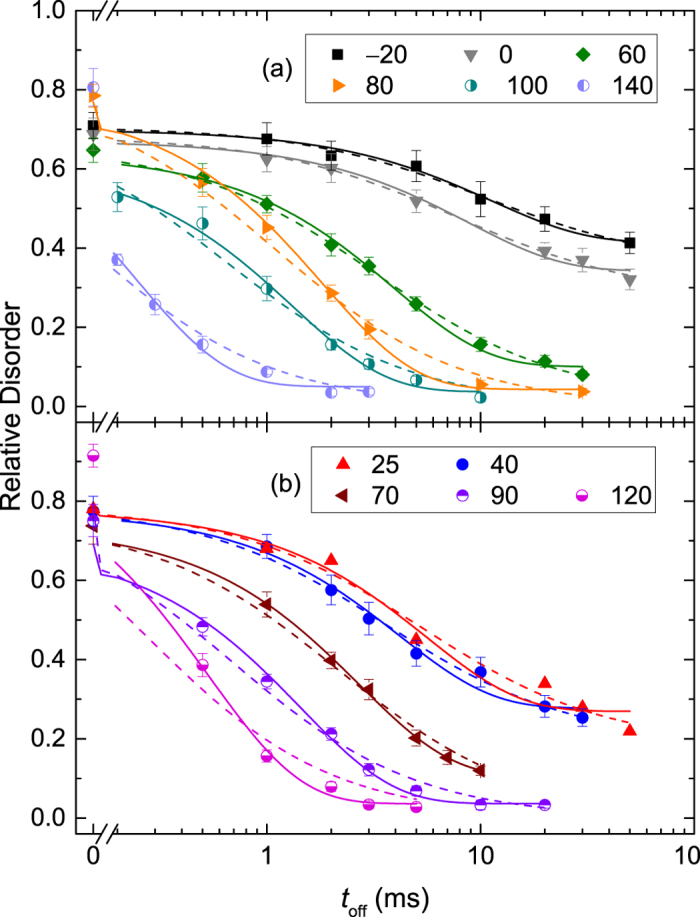
Average relative bulk disorder in Si bombarded at different temperatures (given in legends in units of °C) with a pulsed beam of 500 keV Ar ions with *F*_*on*_ = 1.9 × 10^13^ cm^−2^ s^−1^ and *t*_*on*_ = 1 ms as a function of the passive portion of the beam duty cycle (*t*_*off*_). Results of fitting the data with the first and second order decay equations are shown by solid and dashed lines, respectively. Results are separated into panels for clarity.

Figure 1 shows representative depth profiles of relative disorder for bombardment of Si with continuous (*t*_*off*_ = 0 ms) and pulsed (*t*_*off*_ = 3 and 30 ms) beams at *T*s of 40 and 80 °C. It is seen that, for both *T*s, these depth profiles are bimodal, with the first small peak at the sample surface and the second major peak in the crystal bulk. The bulk peak is centered on ~500 nm, which corresponds to the maximum of the nuclear energy loss profile for 500 keV Ar ions^16^. It is seen from Fig. 1 that the average bulk disorder (*n*) decreases with increasing *t*_*off*_ for both *T*s. This is better illustrated in Fig. 2, which summarizes all the *n(t*_*off*_) dependencies for *T*s from −20 to 140 °C. It is seen from Fig. 2 that, for all the *T*s studied, *n* monotonically decreases with increasing *t*_*off*_. This effect is due to the interaction of mobile defects generated in different pulses and, hence, in different cascades (i.e., inter-cascade defect interaction).

Decay dependencies, as revealed by Fig. 2, are commonly described by either first or second order decay equations in order to estimate decay time constants and, hence, kinetic rates. In a first kinetic order process, the rate is directly proportional to the concentration of interacting species, while in the second order process, the rate is proportional to the square of the concentration. Examples of the first order processes of defect interaction include trapping of interstitials or vacancies at sinks, whereas direct vacancy–interstitial annihilation and the formation of di-vacancies and di-interstitials are examples of the second order processes.

The *n(t*_*off*_) dependencies from Fig. 2 were fitted via the Marquardt-Levenberg algorithm^18^ with first (*n(t*_*off*_) = *n*_∞_ + (*n*(0) − *n*_∞_) exp(−*t*_*off*_/*τ*_1_)) and second order 
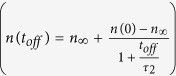
 kinetics equations, represented in Fig. 2 by solid and dashed lines, respectively. Here, *τ*_1,2_ is the characteristic decay time constant, and *n*_∞_ is relative disorder for 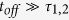. Since a decrease in *n* with increasing *t*_*off*_ is a result of inter-cascade defect interaction, the time constant *τ* reflects inter-cascade defect interaction processes. Below 60 °C, the data is fitted best with the second order decay equation, while the first order decay gives a better fit for all the *n(t*_*off*_) dependencies above 60 °C. Such an evaluation of the kinetic order of *n(t*_*off*_) dependencies was done by comparing *R*-squared values of fits with the first and second order decay equations. In all the cases of different *T*s, however, *R*-squared values were >0.96.

**Figure 3:**
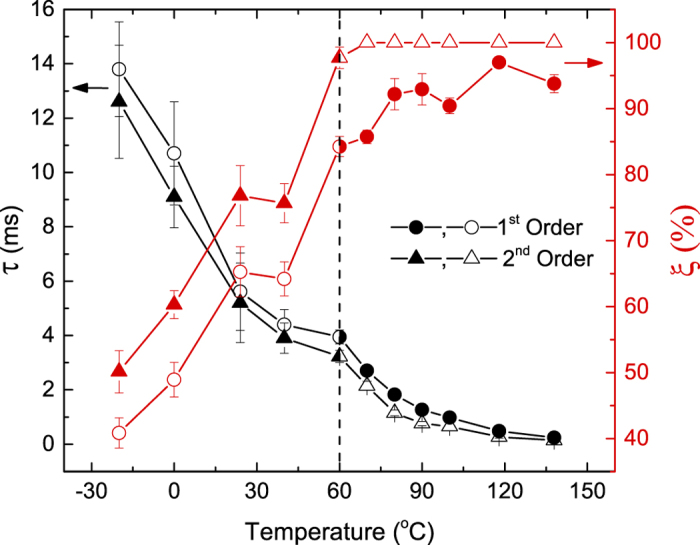
Temperature dependencies of the effective time constant of DA (*τ*, the left axis) and the DA efficiency (*ξ*, the right axis) for fitting with the first (circles) and second (triangles) order decay equations. Solid symbols represent the best fit to the data.

Temperature dependencies of *τ*_1_ and *τ*_2_ are plotted in Fig. 3, revealing a monotonic decrease with increasing *T* and a kink (i.e., a change in the first derivative) at 60 °C in both curves. As expected from the form of first and second order decay equations, *τ*_1_ > *τ*_2_. Also plotted in Fig. 3 is the *T* dependence of the DA efficiency *ξ*_1,2_, which we define as before refs [7, 8]: *ξ* = (*n*(0) − *n*_∞_)/*n*(0). Figure 3 shows that, within experimental errors, *ξ* increases with *T*, reflecting a corresponding decrease in *n*_∞_, which is also clearly seen in *n(t*_*off*_) dependencies of Fig. 2. Above ~60 °C, *ξ*_1_ saturates at ~90%. Note that the apparent saturation of *ξ*_2_ at 100% for higher *T*s is an artifact of inferior fitting with the second order decay equation above 60 °C.

**Figure 4:**
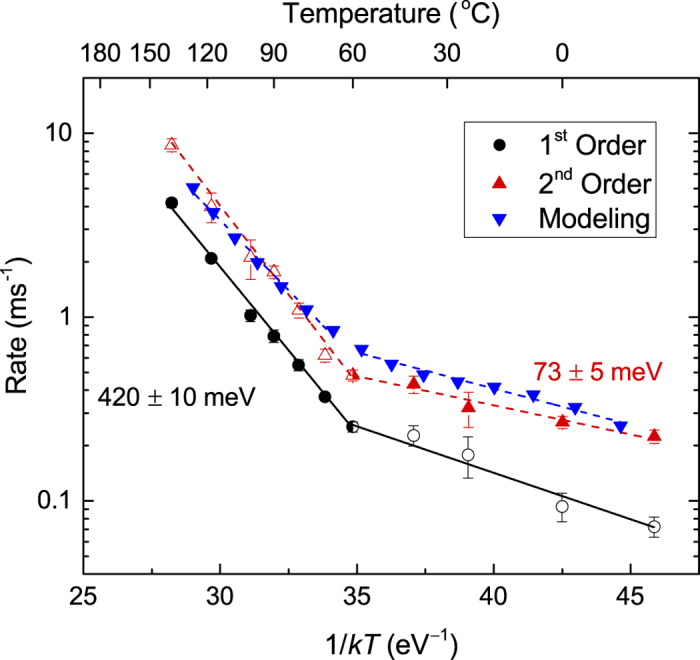
Arrhenius plot of the DA rate. Solid symbols are results of the best fit to experimental *n(t*_*off*_) dependencies, which is the second order decay below 60 °C and the first order decay above 60 °C. Straight lines show results of linear fitting, revealing activation energies of 420 meV and 73 meV above and below 60 °C, respectively. Results of rate theory modeling, described in the text, are also shown.

In Fig. 4, we replot the *τ*_1,2_(*T*) dependencies from Fig. 3 in Arrhenius coordinates, with the DA rates (*k*_1,2_) defined as 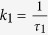 and 
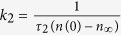
 for the first and second order decay processes, respectively, and with *kT* having the usual meaning. Two Arrhenius regimes are clearly seen in Fig. 4, below and above 60 °C. Fitting of the data to 
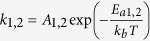
 gives *E*_*a*_s and pre-exponential factors (*A*_1,2_) of *E*_*a*2_ = 73 ± 5 meV and *A*_2_ = (6.1 ± 1.3) × 10^3^ Hz for *T* < 60 °C and *E*_*a*1_ = 420 ± 10 meV and *A*_1_ = (1.43 ± 0.69) × 10^9^ Hz for *T* > 60 °C. Values of *E*_*a*_s obtained by fitting with the first and second order decay equations are comparable. Indeed, fitting data from Fig. 4 with the first order decay equation for *T* < 60 °C and with the second order decay equation for *T* > 60 °C gives *E*_*a*_s and *A*s of *E*_*a*1_ = 110 ± 10 meV and *A*_1_ = (15.3 ± 5.4) × 10^3^ Hz for *T* < 60 °C and *E*_*a*2_ = 440 ± 20 meV and *A*_2_ = (4.05 ± 2.9) × 10^9^ Hz for *T* > 60 °C.

Our experimental data unambiguously reveals the existence of two distinct dominant DA process at *T*s below and above 60 °C, evidenced by (i) the presence of two well-defined Arrhenius regions with vastly different *E*_*a*_s and *A*s (Fig. 4), (ii) a saturation of *ξ* for *T* > 60 °C (Fig. 3), and (iii) the change from the second order to the first order kinetic behavior. The switch from the second kinetic order to the first order behavior at 60 °C points to a change in the dynamics of defect interaction. Our Raman scattering measurements (with 633 nm laser light) of samples irradiated at different *T*s, however, have not revealed any evidence of the change in the damage state at 60 °C. In addition, our transmission electron microscopy analysis has not revealed any differences in the type of damage in Si irradiated with pulsed and continuous beams to the same disorder level.

The two *E*_*a*_s of ~73 and ~420 meV (Fig. 4) are in contrast to much larger *E*_*a*_ values of ~0.7–1.7 eV reported in previous dose rate studies^2^,^3^,^4^. In most of these prior attempts to measure the *E*_*a*_ of DA^2^,^3^,^4^, the dose rate was used as the rate of the kinetic process in the Arrhenius relationship, with the implicit assumption that the dose rate is proportional to the rate of the dominant DA process. The large difference between the *E*_*a*_ values measured in the present work and those in previous dose rate studies^2^,^3^,^4^ could be attributed to the fact that, in the dose rate approach, the *E*_*a*_ is effectively extracted from the *ξ(T*) dependence. As discussed in detail recently^8^, for our choice of *F*_*on*_ and *t*_*on*_, *ξ* is the magnitude of the dose rate effect; i.e., the difference between *n* for continuous beam irradiation with dose rates of *F* = *F*_*on*_ and *F* → 0. Hence, *ξ* reflects the fraction of ballistically-generated Frenkel defects that participate in DA processes for any given *F*_*on*_ rather than the *rate* of defect interaction. In other words, while *τ* reflects the DA rate (i.e., dynamics), *ξ* describes the DA “magnitude”. This is further supported by Fig. 3, revealing qualitatively different *ξ(T*) and *τ(T*) dependencies, indicating that parameters *ξ* and *τ* provide complementary information.

With such a large spread in the *E*_*a*_ values reported previously^2^,^3^,^4^,^5^, the dominant DA processes in Si have remained elusive, with suggestions that the mobile defects in the high-dose regime dominated by inter-cascade DA processes behave differently from the migration of vacancies and interstitials in the low-dose regime of intra-cascade DA effects, commonly monitored by electron paramagnetic resonance (EPR)^11^, positron annihilation spectroscopy (PAS)^12^, and deep level transient spectroscopy (DLTS)^15^. In contrast, our results strongly suggest that, even at relatively high doses and dose rates typical for technologically relevant radiation environments, point defect migration still plays a key role in inter-cascade DA. We first note that an *E*_*a*_ of ~73 meV for *T* < 60 °C agrees with *E*_*a*_s previously associated with the migration of isolated interstitials in Si revealed in DLTS (ref. 15) and PAS (ref. 12) measurements. Similarly, an *E*_*a*_ of 420 meV for *T* > 60 °C agrees with *E*_*a*_s assigned to the migration of neutral vacancies (~0.40–0.46 eV) reported in EPR^11^, PAS^12^, and Raman spectroscopy^13^ measurements. It is also consistent with several theoretical predictions^19^,^20^,^21^,^22^,^23^. We note that charge states of mobile defects are important for low dose irradiation conditions when lattice dopants determine the position of the Fermi level and the defect charge state. In contrast, the present study focuses on the regime of relatively high doses (and, hence, high damage levels) when we are likely dealing with only neutral vacancies and interstitials. Indeed, in the present study, the concentration of radiation-generated (stable) lattice defects largely exceeds the initial dopant concentration, and the material is in a semi-insulating state^8^. This conclusion is further supported by a recent pulsed beam study which showed *τ* to be independent of the doping level for 500 keV Ar ion irradiation of Si at RT^8^.

The dominant role of defect migration in inter-cascade DA is also consistent with a relatively large average separation between the centers of adjacent cascades in every pulse (~72 nm or ~300 atomic spacings). In such cases, inter-cascade defect interaction could not proceed without significant defect migration. For a three-dimensional random walk with a defect moving only a single atomic spacing during each jump, ~200,000 jumps would be necessary for the defect to travel the distance between the centers of two adjacent collision cascades^24^. Hence, while the *E*_*a*_ of defect migration may not be the largest energy barrier a defect on the path to recombination or trapping must overcome, it is a barrier which must be overcome numerous times.

To get insight into the atomistics of defect interaction and to better correlate the *E*_*a*_s measured here with energetic barriers of specific defect migration or interaction processes, we have implemented the rate theory modeling as in refs 25, 26, 27, 28, bench-marked against our experimental data. A successful description of both *n(t*_*off*_) and *τ(T*) dependencies has been obtained with a model considering only the following four processes: (i) ballistic Frenkel pair generation (*I*s and *V*s), calculated with the TRIM code^16^; (ii) the annihilation of *I*s and *V*s at unsaturating sinks (such as point defect clusters), (iii) *V* + *V* → *V*_2_, and (iv) *I* + *V*_2_ → *V*. Here, *I, V*, and *V*_2_ refer to interstitials, vacancies, and divacancies, respectively. The *V* + *I* → ∅ reaction is omitted since it does not affect the balance between *V* and *I*, which is critical for stable damage accumulation within this model. We have also found that adding the *V* + *I* → ∅ reaction to the model has no effect on the resultant *E*_*a*_ values. The equations for *T*-dependent interaction parameters were taken from ref. 28. Migration energies of *V*s and *I*s were set to 400 and 100 meV, respectively, while all other energetic barriers were set to zero. All capture radii were set to 5*λ*, where *λ* is the atomic spacing in Si. The total dose and the dose rate were set to 10^12^ cm^−2^ and 10^13^ cm^−2^ s^−1^, respectively, with a *t*_*on*_ of 1 ms. The attempt frequency and the sink concentration were set to 10^−11^ s^−1^ and 8.5 × 10^16^ cm^3^, respectively, and the bulk disorder (*n*) was represented by the *V*_2_ concentration, as in previous studies^25^,^26^,^27^,^28^.

Results of such rate theory modeling are shown in Fig. 4, along with linear fits (dashed lines) to determine *E*_*a*_s. It is remarkable that, such a relatively simple model is capable of reproducing a rather complex experimental data set: *n(t*_*off*_) dependencies and the Arrhenius behavior of the DA rate. Within this model, an *E*_*a*_ of ~400 meV in the Arrhenius plot of Fig. 4 for *T* > 60 °C indeed corresponds to the *V* migration energy. However, an *E*_*a*_ of ~100 meV for *T* < 60 °C in Fig. 4 actually does not correspond to the *I* migration energy. Instead, it reflects a competition between the two channels for *V* annihilation, one by trapping at sinks and the other via the formation of *V*_2_. While *V*_2_ formation dominates at lower *T*s, *V* annihilation at sinks becomes the rate limiting process at *T* > 60 °C. The much lower *E*_*a*_ for *I* migration results in an *I* migration rate that is orders of magnitude faster than that of *V*s. As a result, *τ* is controlled predominantly by the *V* migration rate in the entire *T* range studied. Hence, modeling also helps us understand the origin of the critical *T* of 60 °C in Fig. 4.

However, this relatively simple model has limitations. It takes into account only a very limited subset of possible defect interactions and ignores possible contributions from interstitial clusters and larger vacancy clusters. This simple model cannot quantitatively describe the full range of the damage buildup up to lattice amorphization as it does not take into account the non-linear processes leading to a super-linear (sigmoidal) damage accumulation at elevated *T*s. This model also does not include nonlinear cascade density effects leading to the formation of thermal and/or displacement spikes^29^. In addition, this model does not predict the switch from the 2nd to the 1st order kinetic behavior at 60 °C. More work is currently needed for the development of more sophisticated and physically realistic models that can describe the entire range of experimental observations, including the damage buildup and defect interaction dynamics under different irradiation conditions. Future experimental work is also needed to study non-linear spike effects on the temperature dependence of defect interaction dynamics in Si.

## Conclusion

In conclusion, we have used the pulsed beam technique to measure the *T*-dependence of the effective time constant of DA in Si bombarded in the *T* range from −20 to 140 °C with 500 keV Ar ions. Results have revealed two well-defined Arrhenius regimes described by activation energies of ~73 and ~420 meV, below and above 60 °C, respectively. A comparison of experimental data with rate theory modeling results suggests that inter-cascade DA in Si proceeds via interstitial and vacancy migration, and the DA rate is limited by processes of vacancy annihilation at sinks and divacancy formation. Our results could have far reaching implications for future studies of radiation defect dynamics as the pulsed-beam method offers a novel way of measuring DA rates and activation energies that could be directly compared with results of predictive modeling. Importantly, this method can be used to measure defect interaction rates under technologically relevant irradiation conditions of pronounced inter-cascade defect interaction in any material, opening the door to studying intricate defect interaction phenomena in materials with the composition across the periodic table.

## Additional Information

**How to cite this article**: Wallace, J. B. *et al*. The role of Frenkel defect diffusion in dynamic annealing in ion-irradiated Si. *Sci. Rep.* **7**, 39754; doi: 10.1038/srep39754 (2017).

**Publisher's note:** Springer Nature remains neutral with regard to jurisdictional claims in published maps and institutional affiliations.
